# Characterization of the *yehUT* Two-Component Regulatory System of *Salmonella enterica* Serovar Typhi and Typhimurium

**DOI:** 10.1371/journal.pone.0084567

**Published:** 2013-12-30

**Authors:** Vanessa K. Wong, Derek J. Pickard, Lars Barquist, Karthikeyan Sivaraman, Andrew J. Page, Peter J. Hart, Mark J. Arends, Kathryn E. Holt, Leanne Kane, Lynda F. Mottram, Louise Ellison, Ruben Bautista, Chris J. McGee, Sally J. Kay, Thomas M. Wileman, Linda J. Kenney, Calman A. MacLennan, Robert A. Kingsley, Gordon Dougan

**Affiliations:** 1 Wellcome Trust Sanger Institute, Wellcome Trust Genome Campus, Hinxton, Cambridge, United Kingdom; 2 University of Cambridge Department of Pathology, Addenbrooke's Hospital, Cambridge, United Kingdom; 3 European Molecular Biology Laboratory, European Bioinformatics Institute (EMBL-EBI), Wellcome Trust Genome Campus, Hinxton, Cambridge, United Kingdom; 4 Positive Bioscience Ltd, Mumbai, India; 5 Medical Research Council Centre for Immune Regulation and Clinical Immunology Service, Institute of Biomedical Research, School of Immunity and Infection, College of Medicine and Dental Sciences, University of Birmingham, Birmingham, United Kingdom; 6 University of Edinburgh Division of Pathology, Edinburgh Cancer Research Centre, Institute of Genetics & Molecular Medicine, Western General Hospital, Edinburgh, United Kingdom; 7 The Department of Biochemistry and Molecular Biology and Bio21 Molecular Science and Biotechnology Institute, The University of Melbourne, Parkville, Australia; 8 Novartis Vaccines Institute for Global Health, Siena, Italy; 9 Mechanobiology Institute, National University of Singapore, T-Lab, Singapore, Singapore; 10 Department of Microbiology and Immunology (M/C 790), University of Illinois at Chicago, Chicago, Illinois, United States of America; Institut National de la Recherche Agronomique, France

## Abstract

Proteins exhibiting hyper-variable sequences within a bacterial pathogen may be associated with host adaptation. Several lineages of the monophyletic pathogen *Salmonella enterica* serovar Typhi (S. Typhi) have accumulated non-synonymous mutations in the putative two-component regulatory system *yehUT*. Consequently we evaluated the function of *yehUT* in *S.* Typhi BRD948 and *S*. Typhimurium ST4/74. Transcriptome analysis identified the *cstA* gene, encoding a carbon starvation protein as the predominantly *yehUT* regulated gene in both these serovars. Deletion of *yehUT* had no detectable effect on the ability of these mutant *Salmonella* to invade cultured epithelial cells (*S.* Typhi and *S*. Typhimurium) or induce colitis in a murine model (*S*. Typhimurium only). Growth, metabolic and antimicrobial susceptibility tests identified no obvious influences of *yehUT* on these phenotypes.

## Introduction

Enteric bacteria have evolved a complex lifestyle involving survival in a variety of niches, including the environment and the intestinal lumen of various animal species. Therefore bacteria must regulate global gene expression in response to their environment, gathering information in part through specialized two-component regulatory systems that sense environmental changes. These systems largely operate by exploiting signal-sensing domains on histidine kinase (HK) sensory proteins linked to cognate response regulators (RR) that can modulate gene expression [1]. The regulons associated with many two-component systems have been defined and in some cases some of their environmental stimuli elucidated [2]. However, others remain poorly characterized. 

Orthologues of the candidate two-component regulatory system *yehUT* are found in almost all enteric bacteria, including plant and animal pathogens [3]. Detectable autophosphorylation of YehU and phosphotransfer to YehT has been shown to occur in *Escherichia coli* [4]. In *E. coli*, *yehUT* regulates *yjiY*, a gene that encoded an inner membrane protein belonging to the carbon starvation protein superfamily. *yehUT* may be involved in the stationary phase control network as YjiY is strongly induced at the onset of this growth phase [3]. However, detailed phenotypic analysis of derivatives harboring mutations in the *yehUT* system identified no other obvious differences with otherwise isogenic wild type *E. coli* [3,5]. However, overexpression of *yehT* in *E. coli* conferred resistance to compounds such as crystal violet, deoxycholate [6], and beta-lactam antibiotics [7]. 

*Salmonella enterica* serovar Typhi (S. Typhi) is a human restricted enteric bacterial pathogen that causes typhoid [8]. *S.* Typhi is a monophyletic pathogen that likely crossed into the human population once thousands of years ago [9]. Thus, all *S.* Typhi have a common ancestor and genetic variation in this pathogen is limited and largely characterized by extensive loss of gene function in the form of pseudogene accumulation [10]. Pseudogenes are defined as genes that are potentially inactivated by mutations including nonsense single nucleotide polymorphisms (SNPs), frame-shifts and truncation by deletion or rearrangement [10-13]. Whole genome sequence of multiple *S.* Typhi isolates has revealed its remarkable clonal nature with very few non-synonymous SNPs [9,10]. However, Holt et al. [10] identified several genes containing more than six substitutions when compared to the *S.* Typhi CT18 genome, deviating from the expected Poisson distribution in terms of the accumulation of non-synonymous SNPs, and these proteins may therefore be under selection. In *S.* Typhi, the *yehUT* operon has accumulated relatively more non-synonymous mutations in a number of lineages of the phylogenetic tree compared to other genes, suggesting this operon is potentially undergoing adaptive evolution [9,10]. These observations stimulated us to conduct a more in depth investigation of the *yehUT* loci in an attempt to identify a phenotype in *Salmonella*. 

## Materials and Methods

### Ethics

Ethical approval for the use of serum samples in this study was granted by the Life and Health Sciences Ethical Review Committee of the University of Birmingham, UK. Informed written consent was obtained from all participants. 

For the mouse experiments wild type C57BL/6 mice 8-10 weeks of age were maintained according to UK Home Office Animals (Scientific Procedures) Act 1986 Amendment Regulations 2012 and the Code of Practice for the housing and care of animals used in scientific procedures. The protocols (Home Office Project licence number: 80/2596) were approved by the Animal Welfare and Ethical Review Body at the Wellcome Trust Sanger Institute. Surgery was performed under isofluorane anaesthesia to eliminate suffering.

### Bacteria used for mutant construction

For safety reasons an attenuated derivative of *S.* Typhi Ty 2 (BRD948 Δ*aroA*, Δ*aroC*, Δ*htrA*) was used throughout as the parental isolate for the construction of mutant derivatives [14]. *S*. Typhimurium ST4/74, originally isolated from a calf with salmonellosis, was used as the parent strain for mutant derivative construction [15].

### Informatics analyses

The domain architecture of YehU/YehT was analyzed using InterPro [16]. Transmembrane alpha helical segments were predicted with the TMHMM webserver [17]. The *yehUT* genes of *S*. Typhi Ty2 (Accession number AE014613) and *S*. Typhimurium SL1344 (Accession number FQ312003) were analyzed using the Genome browser Artemis [18]. The ST4/74 strain is the older designation of SL1344 [19]. The analysis required a completed genome sequence with annotation that is available for SL1344 (Accession number FQ312003), but not strictly for ST4/74. The amino acid sequences of YehU/YehT between *S*. Typhi Ty2 (Accession number AE014613) and *S*. Typhimurium SL1344 (Accession number FQ312003) were compared using ClustalW2 [20,21]. The YehU/YehT protein sequences were also compared with the two-component regulatory system of EnvZ/OmpR using ClustalW2 [20,21]. Non-synonymous SNPs in the *yehUT* genes of S. Typhimurium SL1344 (Accession number FQ312003) were identified by comparison to *S*. Typhi CT18 (Accession number: AL513382) using ClustalW2 [20,21].

### Growth conditions

Experimental analysis determined that *yehU* was expressed during growth in minimal media, pH 7.0, with glucose or acetate as a carbon source (Accession number GSE2456). Therefore, for the SDS-PAGE and Western blot analysis *S.* Typhi, *S*. Typhimurium and their mutant derivatives were grown (where stated) in Luria-Bertani (LB) broth containing different salt concentrations (low salt, 0.09M NaCl; standard, 0.17M NaCl), in SPI-2 inducible conditions as described in [22], or in SPI-2 inducible conditions including either 0.5% glucose or 0.25% acetate [23]. Cultures were shaken at 37°C overnight and standardized to an OD600 of 1.0.

In all other experiments strains were grown in low salt LB broth. All *S.* Typhi cultures were supplemented with a mixture of aromatic amino acids (aro-mix; 4μg/ml phenylalanine, 4μg/ml tryptophan, 1μg/ml paraaminobenzoic acid and 1μg/ml dihydrobenzoic acid) and 4μg/ml tyrosine. Cultures were routinely shaken at 200rpm and incubated overnight at 37°C in a non-baffled flask. All mutant strains were grown with 30μg/ml kanamycin (Roche), 20μg/ml chloramphenicol (Sigma) or 30μg/ml ampicillin selection where appropriate.

### Construction of the Δ*yehUT* mutant derivatives

The Δ*yehUT* mutant derivatives of *S.* Typhi BRD948 and *S*. Typhimurium ST4/74 were constructed using the λ red-recombinase system, described previously [24]. Bacteria were transformed with pSIM18 carrying the λ red-recombinase genes [25]. PCR amplification of the *cat* or *aph* genes using pKD3 or pKD4 (5ng/μl) respectively, with appropriate primers (Table S1 in File S1) that contain the priming sites GTGTAGGCTGGAGCTGCTTCG (forward) and CATATGAATATCCTCCTTAG (reverse), homologous to regions on the template plasmids. *S.* Typhi targeting primers were designed using the Ty2 genome sequence [26] and *S*. Typhimurium targeting primers were designed using the SL1344 genome sequence [27] as references (Table S1 in File S1). PCR conditions used were: 94°C for 2 min; 30 cycles of 94°C for 30s; 55°C for 30s; and 72°C for 2.5 min; final elongation 72°C for 7 min [24]. The PCR product (3-5ng) was transformed into *S.* Typhi pSIM18 and *S*. Typhimurium pSIM18, which had been grown in hygromycin broth to an OD600 of 0.4 and induced to express λ red recombinase with heating to 42°C for 15 minutes. Mutant clones that were able to grow on the appropriate antibiotic selective media were cultured overnight at 37°C.

Phage P22 lysates for *S*. Typhimurium were made on the transformants and used to transfer the antibiotic resistance gene into a fresh *S*. Typhimurium strain background. Plasmid pSIM18 was cured from *S.* Typhi and single colonies that grew on LB with appropriate antibiotic but failed to grow on hygromycin agar were selected as mutant clones. The FRT (FLP recognition target)-flanked antibiotic resistance cassette was then eliminated with pCP20, as previously described [28]. Unless stated otherwise mutations constructed as part of this work were designed to minimize polar effects unless otherwise stated. 

In *S*. Typhimurium, mutations in *yehUT* were complemented by co-transducing wild type *yehUT* gene with a closely linked *aph* gene that conferred kanamycin resistance to transductants, into the chromosome of *S*. Typhimurium **Δ** *yehUT*::*cat* using phage P22. Bacterial colonies that grew on LB with kanamycin and failed to grow on LB with chloramphenicol were selected. PCR was then carried out to confirm that we had directly replaced the *yehUT* deletion with an intact *yehUT* copy (Table S2 in File S1).

In *S.* Typhi, the wild type *yehUT* coding sequence was PCR-amplified using primers (Table S2 in File S1). This DNA fragment was then digested by restriction enzymes XbaI and KpnI and ligated to the low copy plasmid pWKS30 [29]. The resulting vector was introduced into the mutant *S*. Typhi **Δ** *yehUT*::*cat*  by electroporation to produce the complement strain *S*. Typhi **Δ** *yehUT*::*cat* pWKS30:: *yehUT*::*aph*.

### Construction of *yehT*::FLAG

A FLAG epitope tag peptide was added to the end of the *yehT* response regulator using a modification of the standard chromosomal gene disruption protocol [30]. A C-terminal FLAG translational fusion of *yehT* was constructed by PCR amplification of pSUB11 using primers gtaagccgtcgctatctgaaaagtttaaaagaggcgattggcctgGACTACAAAGACCATGACGG (forward) and ccttcggtcgtttctatggcaaaacgatattctaacagtcttttaATATGAATATCCTCCTTAG (reverse) (Table S1 in File S1). This PCR product was transformed into *S.* Typhi BRD948 and *S*. Typhimurium ST4/74 containing pSIM18.

### Western blotting

Cultured bacterial cells were centrifuged and resuspended in 300μl of sterile PBS and immediately mixed with 300μL of Laemmli 4X lysis buffer (5% SDS; 10% 2-mercaptoethanol; 40% glycerol; 0.005% bromophenol blue; 250mM 16:24 Tris HCl pH 6.8). Suspensions were heated at 100°C for 5 minutes. Extracted proteins were separated by SDS-PAGE and visualized by Coomassie-blue staining (Sigma) to ensure equal gel loading of proteins. Bacterial proteins resolved by SDS-PAGE were transferred to a nitrocellulose membrane (Invitrogen) and detected with mouse anti-FLAG M2 monoclonal antibodies conjugated to horseradish peroxidase (HRP) (1:5000 (Sigma)), which were consequently bound to HRP-conjugated rabbit anti-mouse antibodies (1:5000 (Dako)). Detection was performed by chemiluminescence (Amersham ECL system, GE Healthcare)[30]. Proteins of the appropriate parental strain were used as a negative control (*S.* Typhi BRD948 or *S*. Typhimurium ST4/74) and *S*. Typhimurium SL1344 *fliA* FLAG was constructed as described for the *yehT*::FLAG and used as a positive control (27kDa).

### DNA microarray analysis (Accession number GSE50825)

The mRNA transcriptional profiles of *S.* Typhi and *S*. Typhimurium and derivatives harboring Δ*yehU*, Δ*yehT* and Δ*yehUT* mutations were analyzed in at least three individual experiments. Overnight bacterial cultures grown in low salt LB shaking at 37°C were diluted to 1 in 100ml of LB broth and grown to an OD_600_ of 0.3 (mid-log phase). Total RNA of ≈ 1X10^9^ cfu was extracted following treatment with 1mg/ml lysozyme for 5 minutes and purification using the RNeasy mini kit (Qiagen), following the manufacturer’s instructions. 

RNA quantity and quality was analyzed using the 2100 Bioanalyser (Agilent) and 25ng/μl of RNA was used to generate cDNA. This was labeled with a single-colored dye probe (Cy3) and hybridized to the appropriate custom microarrays (Agilent). The arrays were scanned using the DNA high-resolution microarray scanner (Agilent). The raw data was normalized by the RMA method [31] and differentially expressed genes were identified using the “*Limma*” Package in R (Bioconductor) [32]. Those genes with at least two-fold difference in expression and an adjusted p-value of <0.05 (using the Benjamini-Hochberg method) were considered to be differentially expressed.

### qRT-PCR

Genes that were significantly differentially expressed in the DNA microarray experiments were tested using qRT-PCR. Using RNA extracted and purified in the different DNA microarray experiments, cDNA was synthesized using QuantiTect Reverse Transcription kit (Qiagen) with random primers, according to the manufacturer’s instructions. PCR was conducted using 20ng/μl cDNA template in a 15μl reaction, with 0.5μM of each primer set (Table S3 and S4 in File S1) and Taq polymerase mix (Qiagen). PCR conditions used were: 95°C for 2 min; 40 cycles of 94°C for 15s; 60°C for 30s; 72°C for 30s; 95°C for 15s, 60°C for 1 min; and 95°C for 15s (Qiagen). The qRT-PCR was performed with a StepOne Plus machine (Applied Biosystems) using SYBR Green Dye (Qiagen). Fold change in gene expression was normalized to expression of the housekeeping gene *recA* using the delta-delta Ct method [33]. 

### Invasion assay

Human laryngeal epithelial (HEp-2) cells were obtained from American Type Culture Collection (ATCC) 10801 University Boulevard Manassas, VA 20110, USA. The cells were maintained in DMEM-F12/L-Glut/FBS as described [34]. Bacteria for this invasion assay were first grown during the day by shaking in low salt LB at 220 rpm for 6 hours (at 37°C) followed by inoculating 0.5mls of this culture into 45mls fresh low salt LB in a 50ml falcon tube and left to grow statically overnight at 37°C [35]. Confluent monolayers of HEp-2 cells were inoculated at a dose of 10^6^ cfu of bacteria. Infected monolayers were then incubated for 1 hour in a tissue culture incubator at 37°C, washed twice with Phosphate Buffer Saline (PBS), and then overlaid with tissue culture medium supplemented with 100μg/ml gentamicin. After 90 minutes, the cell culture was washed three times with PBS and lysed with 1% Triton X-100 in PBS to release intracellular bacteria. An aliquot of this suspension was used to determine the number of intracellular bacteria by plating serial dilutions onto LB agar plates. The bacterial counts of the control strains were compared with their respective Δ*yehUT* mutant derivatives.

### Vi phage assay

S. Typhi BRD948 and its mutant derivative *S.* Typhi Δ*yehUT* were tested for sensitivity (plaque morphology) and susceptibility (bacterial counts) to Vi phage types II and VII [36]. The strains were grown in 3ml of LB left shaking overnight at 37°C. Molten 0.35% L-agar was cooled to 42°C, and 3ml aliquots were added to Falcon tubes containing 10μl of dilutions of Vi phage type II or VII (from 10^-1^ to 10- ^7^) and 150μl of the bacterial culture; the mixtures were poured immediately onto L-agar plates. The plates were left overnight at 37°C. The next day plaque morphology and count was compared.

### Serum bactericidal assays with human sera

Killing of *S.* Typhi, *S*. Typhi Δ*yehUT*, and *S*. Typhi Δ*yehUT*::*cat* pWKS30::*yehUT*::*aph* and *S*. Typhimurium, *S*. Typhimurium Δ*yehUT* and *S*. Typhimurium Δ*yehUT*::*cat yehUT*::*aph*) with fresh, human serum were assessed as previously described [37]. Bacteria in log growth at a concentration of 1 x 10^7^ cfu/ml were added at a 1:10 dilution to 100% human serum to give a final concentration of 1 x 10^6^ cfu/ml and incubated at 37°C for 180 min on a rocker plate at 20rpm. Sera heat-inactivated at 56°C for 30 min were used as a control. Samples were taken at 45, 90 and 180-minute intervals. The concentration of *Salmonella* was determined by serial dilutions and plating on LB agar. 

### Mouse experiments

#### Acute infection mouse model

Mixed competitive inhibition experiments was performed using groups of ten isofluorane-anaesthetized wild type C57BL/6 mice, which were orally inoculated, with 10^8^ cfu of mixed culture of *S*. Typhimurium Δ*phoN*::*aph* and *S*. Typhimurium Δ*yehUT*::*cat* in 200μl of sterile PBS. The *phoN* gene is dispensible for the intestinal and systemic phases of murine salmonellosis [38]. Therefore it was deemed an appropriate chromosomal insertion site for the *aph* gene required to distinguish the control *S*. Typhimurium from the mutant derivatives [39,40]. All mice were weighed daily and monitored for signs of illness. For bacterial quantification tissue from the mesenteric lymph nodes (MLN), cecum, ileum, spleen and liver from the mice were collected on day 5-post infection and homogenized in 5ml of sterile PBS. Viable bacteria in the tissues were quantified by serial dilutions and plating onto LB agar containing the appropriate antibiotics to select for the *Salmonella*. Statistical tests were performed by the Wilcoxon signed-ranked test using Prism software (GraphPad Software Inc.) on the mixed competitive inhibition data. Differences between data sets where p < 0.05 were considered statistically significant.

#### Streptomycin-pretreated colitis mouse model

A streptomycin experiment was performed using three groups of five mice. Mice were pre-treated with 50mg of streptomycin in 200μl of sterile water 24 hours prior to infection [41]. One group of five mice were naïve and the other two groups of five mice were anaesthetized using isofluorane and orally inoculated with 10^3^ cfu of either *S*. Typhimurium Δ*phoN*::*aph* or *S*. Typhimurium Δ*yehUT*::*cat*, in 200μl of sterile PBS. All mice were weighed daily and monitored for signs of illness. All mice were culled on Day 3 of infection or mice exceeding 20% total weight loss were culled prior to Day 3 in accordance with UK Home Office guidelines. The ceca of the mice were collected, weighed and homogenized in 5ml of sterile PBS for bacterial quantification, qRT-PCR of inflammatory cytokines and pathological examination. Statistical analyses were performed on the data. Bacterial quantification in which viable bacteria was quantified by serial dilutions on LB agar containing the appropriate antibiotics to select for the *Salmonella*.

qRT-PCR of inflammatory cytokines: RNA was isolated from 3mm-long cecal sections using RNeasy Mini kit (Qiagen) according to manufacturer’s instruction. RNA integrity and quantity were then assessed using Agilent 2100 Bioanalyzer (Agilent Technologies). cDNA was then synthesized using QuantiTect Reverse Transcription kit (Qiagen) with random primers, according to the manufacturer’s instructions. All TaqMan qRT-PCR primers and probes were designed by Primer Express 3.0 (Applied Biosystems) to span exon-exon junction to avoid genomic DNA amplification. PCR was conducted using 5ng/μl cDNA template in a 25μl reaction, with 20μM of each cytokine primer set (Table S5 in File S1) and 10μM appropriate probe, according to manufacturer’s instructions (Thermo Scientific). The *Gapdh* gene was used as the endogenous control. The qRT-PCR was performed with a StepOne Plus machine (Applied Biosystems) using ABsolute Blue qRT-PCR Rox mix (Thermo Scientific). Relative gene expression was determined by normalization to *Gapdh* copy numbers [42].

Histopathology: To evaluate intestinal inflammatory disease histopathology, we fixed cecal segments in 4% paraformaldehyde and stained 5 μm-thick paraffin sections in hematoxylin and eosin according to standard protocols. Scoring of intestinal inflammation was performed in a blinded manner by a clinical consultant histopathologist with comparative pathology expertise, as follows: submucosal edema: mild-1, moderate-2, severe-3; submucosal cellular inflammation: mild-1, moderate-2, severe-3; mucosal cellular inflammation: mild-1, moderate-2, severe-3; crypt abscesses: absent-0, present (occasional/mild)-1, present (moderate/severe)-2; mucosal ulceration: absent-0, present (focal/mild)-1, present (moderate/severe)-2.

Statistical Analyses: Statistical tests were performed using the Mann-Whitney U test using U test (http://elegans.som.vcu.edu/~leon/stats/utest.cgi) on the data. Differences between data sets where p < 0.05 were considered statistically significant.

### Phenotyping microarrays (BIOLOG)

Phenotype microarrays (PM) of metabolism of carbon sources (PM 1 to 2), nitrogen sources (PM 3), phosphorus and sulfur sources (PM 4), biosynthetic pathway substrates (PM 5), and peptide nitrogen sources (PM 6 to 8), osmotic/ionic response (PM 9), pH response (PM 10) and bacterial chemical sensitivity (PM 11 to 20) were performed according to manufacturer’s instructions. (Biolog Inc. Hayward, California, USA) [43]. *S.* Typhi BRD948 and *S*. Typhimurium ST4/74 were used as reference strains for comparison with their respective Δ*yehUT* mutants (*S*. Typhi Δ*yehUT* and *S*. Typhimurium Δ*yehUT*). PM micro titer plates were incubated at 37°C for 24 hours in the Omnilog (Biolog Inc.) and each well monitored for redox indictor change representing kinetic respiration. Tests were performed in triplicate and the kinetic data analyzed using Omnilog PM software (Biolog Inc.). Data was exported from the Biolog File Manager, and further analysis was conducted in R. Data was transformed in to “signal values” [44], and the Bioconductor package Limma [32] was used to test for differences in dye reduction. Benjamini-Hochberg corrected p-values were used to determine statistical significance, controlling for a false discovery rate of 5%.

Minimum inhibitory concentrations (MICs) of the ∆*yehUT* mutants of *S.* Typhi and *S*. Typhimurium were determined using Epsilometer test (E test) strips against antibiotics amoxicillin, tetracycline, ciprofloxacin, ceftriaxone, and meropenem (Oxoid, Hampshire, UK), trimethoprim-sulfamethoxazole and azithromycin (BioMérieux, SA, Marcy-L’Etoile, France). Semi-confluent growth was achieved using 0.5 McFarland organism bacterial suspensions [45] on Iso-Sensitest agar (Oxoid, Hampshire, UK) after 24 hours incubation aerobically at 37°C. Since the mutant strains were not clinical isolates parental strains were used as the controls to see if the deletion of *yehUT* had an effect on antibiotic susceptibility.

## Results

### Informatics analysis of the YehUT system

**Figure 1:**
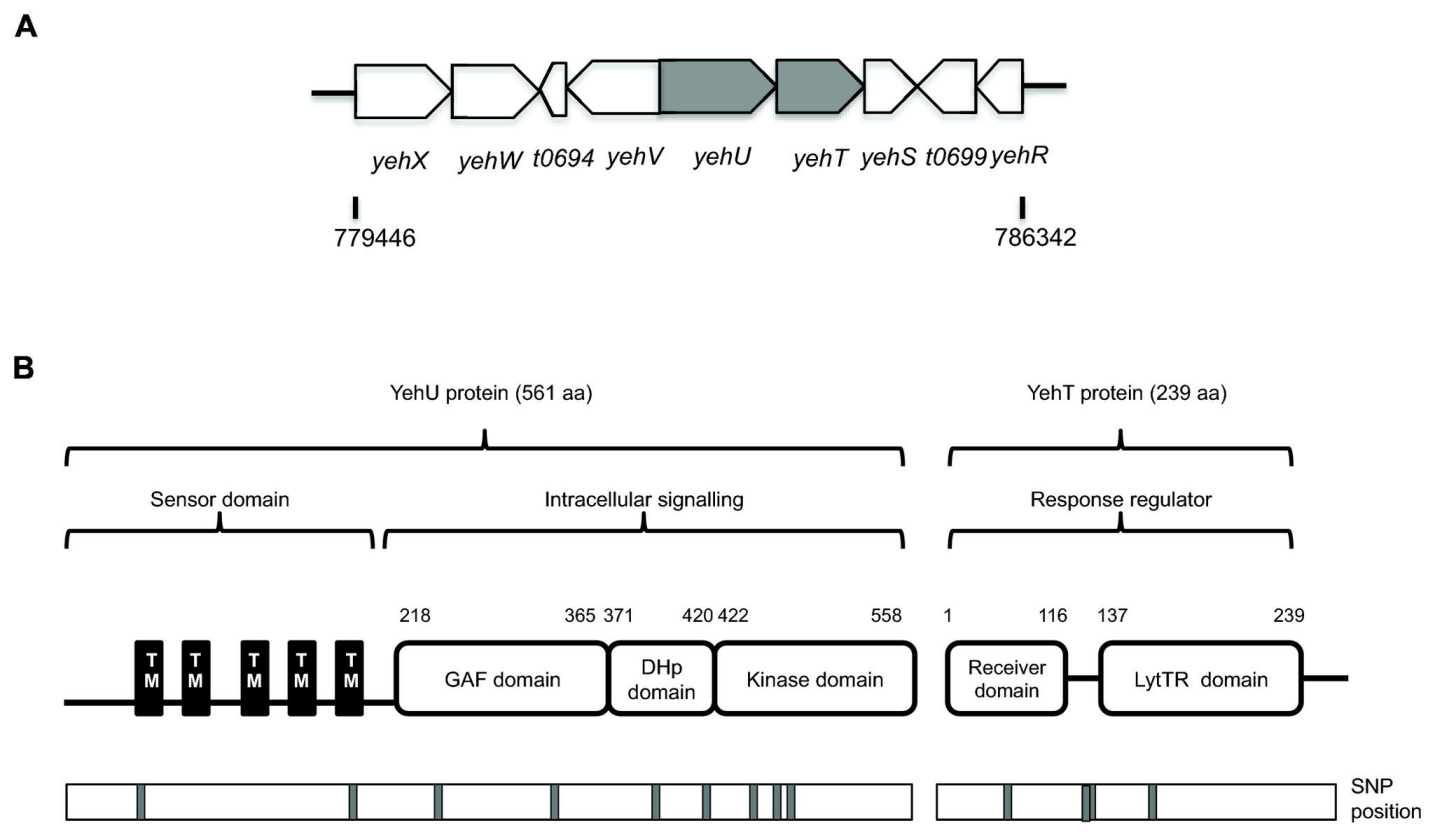
A The *yehUT* operon with surrounding genes. The operon is 2349bp in length and located between 782210 to 784611 bp in *S*. Typhi Ty2 (Accession number AE014613) and between c2251460 to c253861 bp in *S*. Typhimurium SL1344 (Accession number FQ312003). The surrounding genes include *yehV*, *t0694*, *yehW*, *yehX* (upstream of *yehU*) and *yehS, t0699* and *yehR* (downstream from *yehT*). **B The domain organisation of the YehU and YehT proteins**. YehU protein comprises of five transmembrane (TM) alpha helical segments predicted using TMHMM webserver [17]. The intracellular signalling component consists of a GAF domain, a DHp (dimerization/histidine-containing phosphotransfer) domain that is connected to an ATP (adenosine triphosphate)-dependent Kinase domain. YehT protein consists of a Receiver domain and a LyTR-homologous DNA binding domain [16]. The phosphorylation sites are indicated (H, Histidine; D, Aspartate). The SNP positions identified in *yehU* and *yehT* genes are indicated below the proteins by grey boxes [10].

In *S*. Typhi Ty2 the *yehU* and the *yehT* genes are encoded on a 2,349 bp long fragment with a 4 bp overlap between the *yehU* and *yehT* open reading frames and are likely part of an operon. Neighboring genes include *yehV*, *yehW*, and *yehX* upstream of *yehU*, and *yehS* and *yehR* downstream from *yehT*. The genes *yehX, yehW, yehU, yehT* and *yehS* are located on the forward strand, whilst *t0694, yehV, t0699* and *yehR* lie on the reverse strand (Figure 1) [26,46]. The function of these genes is not currently known, although our sequence comparison-using Interpro indicated that *yehV* and *yehS* encode a putative transcriptional regulator and a conserved hypothetical protein, respectively [16]. 

The domain architecture of YehU and YehT was predicted from a consensus definition from Interpro [16]. These results predicted four domains in YehU (sensor, GAF, histidine phosphotransfer (DHp) and kinase domains) and two in YehT, (receiver and cell envelope-related transcriptional attenuator (LytR) domains) (Figure 1). The sensor domain is predicted to contain at least 5 transmembrane helices [17]. GAF domains are so-called because they are found in cGMP-specific phosphodiesterases, adenylyl cyclases and transcriptional activators, including formate hydrogen lyase system activators (FhlA), and although their precise function is not clear they may be involved in ligand binding and protein-protein interaction [47]. The LytTR domain named after the *Bacillus subtillus* LytT and *Staphylococcus aureus* LytR response regulators contains a specific DNA-binding motif and is commonly found in a number of bacterial transcriptional regulators [48]. The YehU/YehT system was compared to EnvZ/OmpR and revealed that YehU does not have a histidine residue at position 243 as in the sensor kinase EnvZ [49].

### YehT expression in different media

**Figure 2:**
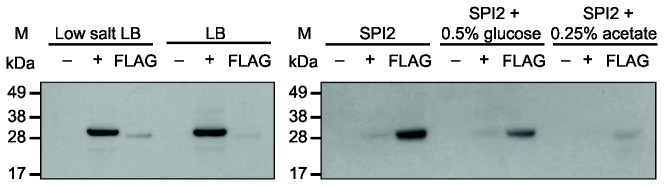
SDS-PAGE and western blot analysis of epitope-tagged YehT protein from *S*. **Typhi BRD948**. Proteins from whole cell bacterial lysates were transferred after electrophoretic separation in a 12% SDS-PAGE gel (Invitrogen) onto a nitrocelluose membrane (Invitrogen) and probed with anti-FLAG M2 monoclonal antibodies (Sigma). Lanes: (M) protein molecular weight markers (SeeBlue Plus2 Pre-stained standard; Invitrogen); (-) proteins extracted from *S*. Typhi BRD948 (negative control); (+) proteins extracted from *S*. Typhimurium SL1344 Δ*fliA* (positive control, 27.4kDa); FLAG proteins extracted from *S*. Typhi Δ*yehT*-tagged (27.4kDa). Bacteria was grown in low salt LB; normal salt LB; SPI-2 inducing; SPI-2 + 0.5% glucose; SPI-2 + 0.25% acetate overnight, shaking at 37°C.

**Figure 3:**
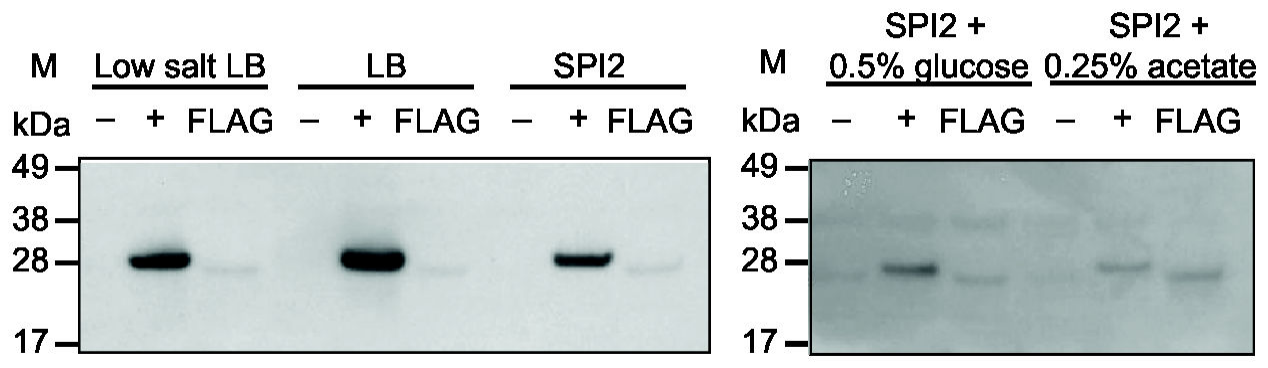
SDS-PAGE and western blot analysis of epitope-tagged YehT protein from *S*. **Typhimurium ST4/74**. Proteins from whole cell bacterial lysates were transferred after electrophoretic separation in a 12% SDS-PAGE gel (Invitrogen) onto a nitrocelluose membrane (Invitrogen) and probed with anti-FLAG M2 monoclonal antibodies (Sigma). Lanes: (M) protein molecular weight markers (SeeBlue Plus2 Pre-stained standard; Invitrogen); (-) proteins extracted from *S*. Typhimurium ST4/74 (negative control); (+) proteins extracted from *S*. Typhimurium SL1344 Δ*fliA* (positive control; 27.4kDa); FLAG proteins extracted from *S*. Typhimurium Δ*yehT*-tagged (27.4kDa). Bacteria was grown in low salt LB; normal salt LB; SPI-2 inducing; SPI-2 + 0.5% glucose; SPI-2 + 0.25% acetate overnight, shaking at 37°C.

In order to study the function of the YehUT system we initially determined the optimal conditions in which the YehT protein of *S.* Typhi and *S*. Typhimurium was expressed. To this end a *S.* Typhi and *S*. Typhimurium derivative was constructed that directed the expression of a YehT fusion protein fused at the C-terminal with the epitope-tag FLAG. Whole protein preparations from these derivatives cultured in five alternative media indicated that YehT was expressed in all conditions. In *S.* Typhi, YehT was expressed at relatively higher level in low salt LB (0.09M NaCl) than standard LB (0.17M NaCl). In a further experiment, expression was also slightly higher in a minimal medium designed to induce SPI-2, compared to the same medium additionally containing either glucose or acetate (Figure 2). However, in *S*. Typhimurium the overall level of expression of YehT was relatively low in all five culture media (Figure 3). Therefore, low salt LB was selected as the best growth media for determination of *yehUT* dependent transcription analysis and elucidation of the phenotype of Δ*yehUT* for both *S.* Typhi and *S*. Typhimurium. 

### Identification of *yehUT* regulated genes

In order to elucidate the role of *yehUT* on global gene expression, we performed transcriptome profiling based on microarray analysis of mRNA purified from *S.* Typhi and otherwise isogenic mutant derivatives harboring non-polar deletions of *yehUT* or either one of these two genes*. S.* Typhi and all mutant derivatives, including Δ*yehUT S.* Typhi grew at comparable rates (data not shown). Analysis of differentially expressed genes relative to the average expression level of each gene across all arrays was performed and genes that showed a statistically significant change in expression in mutant compared to *S.* Typhi control strain were identified (Accession number GSE50825) [32]. The differentially expressed genes were grouped into functional classes based on genome annotation [50].. Transcriptome analysis of the *S.* Typhi transcriptome compared to the Δ*yehU*, Δ*yehT* and Δ*yehUT* mutant derivatives provided evidence of relative down regulation in each of the mutants of three genes: *cstA1* (*t4582*), encoding a carbon starvation protein, and *t4581* and *t4580* annotated as conserved hypotheticals (Table 1).

**Table 1:** DNA microarray analysis of *S*. Typhi Δ*yehUT* compared to *S*. Typhi *BRD948*, grown in low salt LB at mid-log phase.

**Genes**	**Gene ID**	**Putative identification of NCBI database**	**Functional category**	**Log_2_ fold change (FC)**	**Adjusted p-value**
*yehU* mutant					
*cstA1*	t4582	carbon starvation protein	I	-6.517056711	4.24E-07
-	t4581	hypothetical protein t4581	III	-3.984595019	3.42E-04
-	t4580	GTP-binding protein YjiA	III	-2.282197911	4.17E-02
*yehT* mutant					
*cstA1*	t4582	carbon starvation protein	I	-6.36E+00	5.44E-07
-	t4581	hypothetical protein t4581	III	-3.91E+00	4.12E-04
-	t4580	GTP-binding protein YjiA	III	-2.41E+00	3.50E-02
*yehUT* mutant					
*cstA1*	t4582	carbon starvation protein	I	-6.47E+00	4.56E-07
-	t4581	hypothetical protein t4581	III	-3.68E+00	4.95E-04
-	t4221	AraC family transcriptional regulator	V	-1.94E+00	1.45E-02
-	t4220	GerE family regulatory protein	V	-1.55E+00	1.62E-02
*spaM*	t2795	virulence-associated secretory protein	I	-1.52E+00	1.62E-02
-	t4580	GTP-binding protein YjiA	III	-2.32E+00	2.04E-02
*spaN*	t2794	antigen presentation protein SpaN	I	-1.57E+00	2.23E-02
*spaI*	t2796	ATP synthase SpaI	I	-1.46E+00	2.23E-02
*pipB*	t1830	hypothetical protein t1830	I	-1.54E+00	2.61E-02
-	t1038	hypothetical protein t1038	II	1.03E+00	4.21E-02
*yiiD*	t3592	Acetyltransferase	IV	-8.89E-01	4.21E-02
*ppc*	t3505	phosphoenolpyruvate carboxylase	VII	-1.25E+00	4.21E-02
*Nth*	t1321	endonuclease III	VI	1.26E+00	4.21E-02
-	t3408	hypothetical protein t3408	VIII	-9.93E-01	4.60E-02
*sopE*	t4303	invasion-associated secreted protein	VIII	-1.92E+00	4.96E-02

Differential expression profiles were represented by a log_2_ fold change (FC) value – a positive value indicates the gene is up regulated in the mutant strain compared to *S.* Typhi; a negative value indicates the gene is down regulated in the mutant strain compared to . Key genes identified based on an adjusted p-value of <0.05 using the Benjamini-Hochberg method [32]. Gene annotation was performed using the *S.* Typhi Ty2 database at NCBI (http://www.ncbi.nlm.nih.gov/; Accession number AE014613). Functional categories as annotated in the *S.* Typhi CT18 genome (Accession number AL513382) Code: I – pathogenicity/adaptation; II – hypothetical (unknown function); III – conserved hypothetical; IV – central/intermediary metabolism; V – regulators; cyan VI – degradation of macromolecules; VII energy metabolism; VIII – phage-related functions/IS elements.

The *cstA1* gene was the most differentially expressed in all mutant derivatives compared to *S.* Typhi. This gene is located in the same chromosomal region as *t4581* and *t4580* on the reverse strand of the *S.* Typhi Ty2 genome: *cstA1* (*t4582*) is situated at c4734842 bp to c4736992 bp, with *t4581* 95 bp and *t4580* 309 bp downstream, respectively [18]. Previous RNA-sequencing data has shown that these genes are transcribed *in vitro* at 37°C during mid-log phase [51]. The *cstA1* gene was identified as a putative carbon starvation protein using the NCBI database [52,53]. Furthermore, the gene is predicted to encode a membrane protein involved in peptide utilization to avoid carbon starvation as the bacterium enters stationary phase [54]. This protein appears to be conserved in the *Enterobacteriaceae*, including *Salmonella* species [54]. *cstA* has been described as a cAMP-CRP (cyclic adenosine monophosphate-catabolite regulation protein) dependent gene in *E. coli* when the concentration of cAMP rises during carbon starvation activating the gene [55]. 

Both NCBI and Pfam databases identified *t4581* as encoding a hypothetical protein of unknown function, although structural modeling suggested a domain that might bind nucleic acids [52-54]. Gene *t4580* is predicted to encode a putative GTP-binding protein (YjiA), using NCBI [52,53]. In *E. coli* the crystal structure supports a GTP-dependent function, but the biological role of the protein remains unclear [56].

Transcriptome analysis of the *S.* Typhi Δ*yehUT* mutant displayed not only dysregulation of *cstA1*, *t4580* and *t4581*, but also an additional 12 genes: ten genes were down regulated and two up regulated (Table 1). These genes were not differentially expressed in either of the single mutants. There was relative down regulation of a number of Salmonella Pathogenicity Island (SPI) associated genes, including *spaM*, *spaN*, and *spaI* located on SPI-1, *pipB* situated in SPI-5, and the SPI-7 encoded gene *sopE* [57]. Other down regulated genes included *t4221*, a member of the AraC family transcriptional regulator family and *t4220*, a GerE family regulatory protein. Metabolic genes relatively down regulated in the *S.* Typhi Δ*yehUT* mutant included *ppc*, which encodes phosphoenolpyruvate carboxylase, an enzyme involved in the oxidation of carbohydrates via the tricarboxylic acid cycle [58] and *yiiD*, a galactoside O-acetyltransferase, which in *E. coli* may assist cellular detoxification by acetylating non-metabolizable pyranosides and thereby preventing their reentry into the cell [59]. The two genes that were up regulated in the double mutant were the *nth* gene, which encodes endonuclease III involved in the DNA repair system [60,61] and *t1038*, which encodes an unknown hypothetical protein.

**Figure 4:**
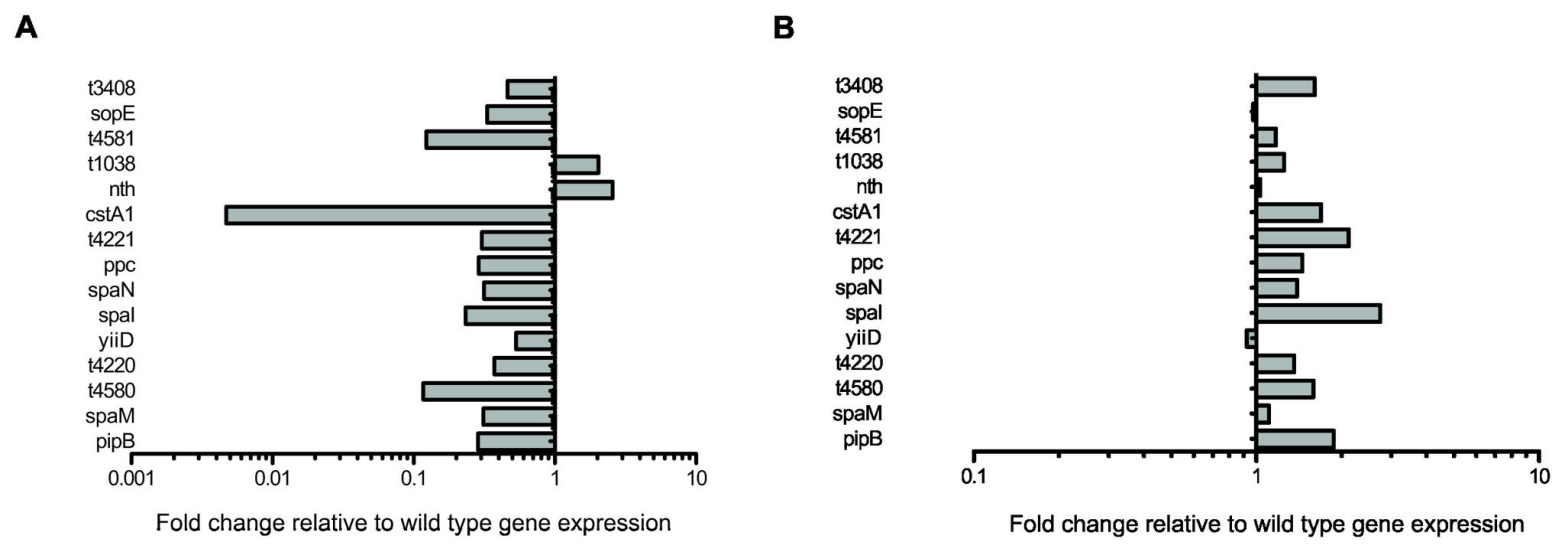
qRT-PCR analyses of differentially expressed genes of the S. **Typhi Δ*yehUT* mutant**. A The relative expression levels of the 15 genes found to be differentially expressed on microarray analysis of the S. Typhi Δ*yehUT* mutant when compared to *S*. Typhi in low salt LB, at mid-log phase, at 37°C (Table S6 in File S1). The 15 genes examined are listed on the *y*-axis. Grey boxes < 1.0 indicates down regulated genes and grey boxes >1.0 indicates up regulated genes relative to *S*. Typhi. B qRT-PCR analysis showing relative expression levels of genes after complementation of the Δ*yehUT* mutant compared to *S*. Typhi (Table S6 in File S1).

The differential expression of several genes, including *cstA1*, *t4581*, *t4580*, *spaM*, *spaN*, *spaI* and *sopE*, were validated using qRT-PCR. The overall pattern of up and down regulation of genes was identical to the microarray results (Table S6 in File S1; Figure 4a). Complementation of the Δ*yehUT* mutant with plasmid-encoded *yehUT* restored expression of the genes, although many of the genes were then relatively over-expressed, which is most likely a result of the high copy number of plasmids containing *yehUT* (Table S6 in File S1; Figure 4b). 

### Phenotypic analysis of the *yehUT* deletion mutant derivatives of *S.* Typhi

S. Typhi BRD948 and its Δ*yehUT* mutant derivative were phenotypically analyzed using serum bactericidal assays (Graph B in Figure S1), tissue invasion assays with Hep2 cells (Graph A in Figure S2), and a Vi phage sensitivity assay (Figure S3), but no significant differences were observed. There was no metabolic phenotype identified for *S.* Typhi Δ*yehUT* using phenotypic Biolog microarrays, which monitors a range of different growth conditions, including various substrates, ionic concentrations (pHs) and osmolarities. Furthermore, no difference in MIC was observed between *S.* Typhi and the Δ*yehUT* mutant derivative when tested against a range of antibiotics commonly used to treat *Salmonella* infections, including amoxicillin, ceftriaxone, ciprofloxacin, trimethoprim-sulfamethaxole, meropenem and azithromycin.

### Properties of a *yehUT* deletion mutant derivative of *S*. Typhimurium

The two-component regulatory system *yehUT* is also present in *S*. Typhimurium which allowed us to use this serovar for a comparison against *S.* Typhi and as a surrogate *in vivo* model of typhoid fever. Informatics analysis of the *S.* Typhimurium SL1344 genome identified one non-synonymous SNP in *yehT* and four non-synonymous SNPs in *yehU* compared to *S*. Typhi CT18 (Table S7 in File S1) [18]. The consensus encoded polypeptides are almost identical in both serovars with only four amino acid differences in YehU and two amino acid difference in YehT identified between *S*. Typhi Ty2 and *S*. Typhimurium SL1344, with no difference observed between predicted protein structures [20,21,54]. 

**Figure 5:**
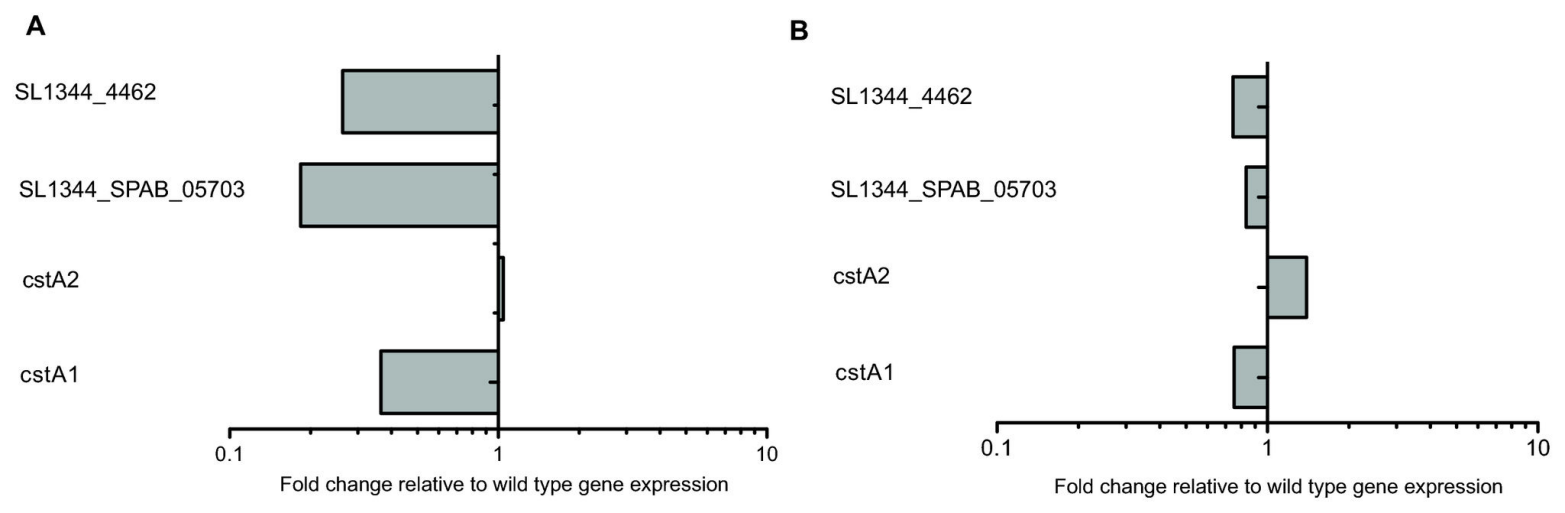
qRT-PCR analyses of differentially expressed genes of the S. **Typhimurium Δ*yehUT* mutant**. A The relative expression levels of the 4 genes found to be differentially expressed on microarray analysis of the S. Typhimurium Δ*yehUT* mutant when compared to *S*. Typhimurium. in low salt LB, at mid-log phase, at 37°C (Table S6 in File S1). The 4 genes examined are listed on the *y*-axis. Grey boxes < 1.0 indicates down regulated genes and grey boxes >1.0 indicates up regulated genes relative to *S*. Typhimurium. B qRT-PCR analysis showing relative expression levels of genes after complementation of the Δ*yehUT* mutant compared to *S*. Typhimurium.(Table S6 in File S1).

Transcriptome analysis of mRNA prepared from *S*. Typhimurium and the *yehUT* mutant derivatives, provided evidence for the comparative down regulation of the same four genes identified in similar experiments with *S*. Typhi: *cstA1* (*SL1344_4463*), *cstA2* (*SL1344_0588*) and hypothetical proteins *SL1344_4462* and *SL1344_SPAB_05703* (Accession number GSE50825) (Table 2). Three out of the four genes (*cstA1*, *SL1344_4462* and *SL1344_SPAB_05703*) were confirmed by qRT-PCR to be relatively down regulated in the Δ*yehUT* mutant (Table S6 in File S1; Figure 5a). Expression of these genes was restored by complementation of the Δ*yehUT* mutant (Table S6 in File S1; Figure 5b). On further analysis these genes are found in the same region in the genome with the *cstA1* gene located at c4808148bp to c4810298 bp on the reverse strand, *SL1344_4462* situated 95bp downstream of *cstA1* on the same strand and *SL1344_SPAB_05703* overlapping upstream of *cstA1* on the forward strand (4810246 bp to 4810350 bp) [18]. Gene *SL1344_4462*, like *t4581* of *S.* Typhi, is predicted to bind nucleic acids. However, the function of *SL1344_SPAB-05703* is unknown [54]. The second *cstA* gene (*cstA2*) was not differentially expressed on qRT-PCR.

**Table 2:** DNA microarray analysis of *S*. Typhimurium Δ*yehUT* mutants compared to *S*. Typhimurium, grown in low salt LB at mid-log phase.

**Genes**	**Gene ID**	**Putative identification of NCBI database**	**Functional category**	**Log_2_ FC**	**Adjusted p value**
*yehU* mutant					
*cstA1*	SL1344_4463	carbon starvation protein	I	-7.36E+00	1.06E-09
-	-	hypothetical protein SPAB_05703	-	-3.82E+00	1.24E-06
-	SL1344_4462	hypothetical protein STY4889	III	-3.06E+00	3.45E-06
*cstA2*	SL1344_0588	hypothetical protein SPAB_02963 (carbon starvation protein)	II	-2.89E+00	9.25E-03
*yehT* mutant					
*cstA1*	SL1344_4463	carbon starvation protein	I	-7.33E+00	5.49E-10
-	-	hypothetical protein SPAB_05703	-	-3.79E+00	1.32E-06
-	SL1344_4462	hypothetical protein STY4889	III	-2.94E+00	5.16E-06
*cstA2*	SL1344_0588	hypothetical protein SPAB_02963 (carbon starvation protein)	II	-3.58E+00	1.25E-03
*yehUT* mutant					
*cstA1*	SL1344_4463	carbon starvation protein	I	-7.66E+00	4.23E-10
-	-	hypothetical protein SPAB_05703	-	-3.94E+00	8.86E-07
-	SL1344_4462	hypothetical protein STY4889	III	-2.68E+00	1.33E-05
*cstA2*	SL1344_0588	hypothetical protein SPAB_02963 (carbon starvation protein)	II	-3.47E+00	1.70E-03

Differential expression profiles were represented by a log_2_ fold change (FC) value – a positive value indicates the gene is up regulated in the mutant strain compared to *S*. Typhimurium; a negative value indicates the gene is down regulated in the mutant strain compared to *S*. Typhimurium. Key genes identified based on an adjusted p-value of <0.05 using the Benjamini-Hochberg method [32]. Gene annotation was performed using the *S*. Typhimurium SL1344 genome (Accession number FQ312003) [46]. Functional categories: I – pathogenicity/adaptation; II – hypothetical (unknown function); III – conserved hypothetical; IV – central/intermediary metabolism; V – regulators; cyan VI – degradation of macromolecules; VII energy metabolism; VIII – phage-related functions/IS elements.

For *S*. Typhimurium and its ∆*yehUT* mutant derivative a range of virulence experiments were performed including serum bactericidal assays (Graph A in Figure S1), tissue invasion assays (Graph B in Figure S2), acute infection mouse model (Figure S4) and streptomycin-treated colitis mouse model (Figure S5-S7). No difference was observed between these isolates. There was also no metabolic phenotype identified for *S*. Typhimurium Δ*yehUT* using phenotypic Biolog microarrays or E-tests against antibiotics amoxicillin, ceftriaxone, ciprofloxacin, trimethoprim-sulfamethaxole, meropenem, azithromycin and tetracycline.

## Discussion

S. Typhi is a Gram-negative organism that causes the disease typhoid fever and is spread fecal-orally by ingestion of contaminated food or water [8]. Unlike most *S. enterica* serovars, *S.* Typhi is a human restricted pathogen that entered the human population once relatively recently. Thus, all *S.* Typhi can be mapped phylogenetically back to a common ancestor and the genomes of collections of *S.* Typhi exhibit limited genetic variation [10]. That the *yehUT* operon of *S.* Typhi was found to contain an unusually high number of SNPs compared to the rest of the genome was therefore of potential significance in the evolution of this pathogen [10]. In addition, large scale transposon mutagenesis (TraDIS) data obtained by investigation of *S*. Typhimurium mutant survival during infection of mice, chickens, pigs and cattle showed that a single insertion in the *yehT* gene had a significant attenuation in calves (www-tradis.vet.cam.ac.uk). Consequently we combined transcriptome analysis and phenotypic assays to gain a better understanding of this system in *Salmonella*.

The transcriptome analysis for both *S.* Typhi and *S*. Typhimurium containing Δ*yehU*, Δ*yehT* or Δ*yehUT* revealed relative down regulation of *cstA1* associated with the response to carbon starvation along with two neighboring hypothetical genes: *t4580* and *t4581* of *S.* Typhi; *SL1344_4462*, and *SL1344_SPAB_05703* of *S*. Typhimurium. These three genes are situated within the same region of the genome of both serovars. The *cstA* gene was previously shown in *E. coli* to be positively regulated by the cyclic AMP (cAMP) and cAMP receptor protein (CRP) complex and to be involved in peptide utilization during carbon starvation [55,62,63]. In *E. coli*, expression was increased upon exhaustion of succinate, glycerol, or glucose from the medium, or upon entering stationary phase in LB [62]. CstA has been implicated as a peptide transporter [63], supported by sequence analysis that revealed the CstA protein consists of 701 amino acids and a hydrophobicity profile suggestive of an integral membrane protein [64]. A CstA homologue YjiY (>96% identity) was also up regulated when *yehT* was over expressed in *E. coli* [3]. Furthermore, YjiY, like CstA, may take part in amino acid/peptide utilization [3]. The remaining 31 genes differentially expressed in the *E. coli* study did not match the genes differentially expressed in *Salmonella*. However, Kraxenberger et al. [3] utilized over expression of the *yehT* gene compared to our mutant derivatives, which may account for at least some of the difference in gene expression profiles.

S. Typhi Δ*yehUT* exhibited differential expression of a further 12 genes compared to the ∆*yehU* and ∆*yehT* mutant derivatives, including genes involved in metabolism (*yiiD, ppc*) pathogenicity (*spaM, spaN, spaI, pipB*) and regulation (*AraC, GerE*). This suggested that *yehUT* might modulate metabolism or virulence functions of *S.* Typhi; hence we went on to test the mutant in various biochemical and virulence assays. The difference in gene expression observed between the derivatives harboring individual and double gene deletions in *S.* Typhi might be indicative of cross-regulation between *yehUT* and another two-component regulatory system compensating for the lone loss of the sensor kinase or response regulator in the *yehUT* system [65,66]. 

An independent study reported relative dysregulation of 26 genes in an *S.* Typhi GIFU10007Δ*yehT* mutant derivative under hypotonic conditions [67]. This dysregulation is somewhat more extensive than the three genes (*cstA1, t4580, t4581*) we identified using a Δ*yehT* mutant derivative of *S.* Typhi BRD948. The number of genes differentially expressed (26 genes) is more compatible with the analysis obtained from our Δ*yehUT* mutant (15 genes), with four genes (*spaN, spaM, spaI, pipB*) apparently dysregulated in both studies. It is noteworthy that the *cstA1* gene was not differentially expressed to a statistically significant level in their study and yet was the top dysregulated gene here in both *S.* Typhi and *S*. Typhimurium *yehUT* mutant derivatives under isotonic conditions. These differences in transcriptome profiling of *yehUT* may be due to differences in the experimental conditions or to the use of different parental strains.

Genetic regulation of the *yehUT* operon is not well characterized. Differential regulation of *yehU* or *yehT* was not seen in a previous study in which we identified genes by microarray and RNA-seq of *S.* Typhi BRD948 that exhibited OmpR-dependent transcription, during mid log phase, in LB media [68]. However, another study showed that OmpR positively regulated expression of *yehUT* in *S.* Typhi GIFU10007 at low osmolarity [67]. One explanation for this discrepancy is that OmpR only induces *yehUT* expression when the osmolarity is lowered. 

It has been proposed that YehU activates YehT by phosphorylation of a conserved histidine residue at position 382 [3]. In *E. coli* mutations in the putative histidine residue of YehU involved in phosphotransfer (position 382) and YehT (position 54) impacted expression of *yjiY*, a component of the *yehUT* regulon, suggesting an important role for phosphorylation at these sites [3]. A previous study has shown that autophosphorylation of YehU and phosphotransfer to YehT were barely detectable under all conditions tested [4]. However, YehU was shown to have no identifiable F box, which is a component of the histidine protein kinase domain involved in ATP binding, and probably also catalysis and phosphotransfer. This property may explain this reduction in phosphorylation [69]. In silico analysis predicted a histidine phosphotransfer (DHp) domain component of YehU, but the conserved histidine residue at 382 was not seen in second sensor kinase EnvZ on alignment comparison suggesting that these regulators have distinct mechanisms of signal transduction.

There are two copies of *cstA* gene homologues with a comparable identity of 61.9% in the genomes of both *S.* Typhi and *S*. Typhimurium serovars: *cstA1* (*S.* Typhi *t4582*; *S*. Typhimurium *SL1344_4463*), which was differentially expressed in our microarray analysis and *cstA2* (*S.* Typhi *t2268*; *S*. Typhimurium *SL1344_0588*), which was confirmed not to be dysregulated [20,21]. In a previous study we demonstrated that OmpR negatively regulates *cstA2* in both *S.* Typhi and *S*. Typhimurium, using DNA microarray analysis, but exerts no effect on *cstA1* [68]. A clearer understanding of the regulatory networks of both *cstA1* and *cstA2* may provide clues to how *Salmonella* is continually fine tuning its adaption to its host and environment.

Other than within the transcriptome profile, we were unable to identify a phenotype for *S*. Typhi or *S*. Typhimurium harboring mutations in *yehUT*. Despite the transcriptome analysis of *S.* Typhi *ΔyehUT* revealing dysregulation of a number of SPI genes *yehUT* mutant derivatives were able to invade cultured epithelial cells at rates comparable to control and showed no difference the mouse infection models compared to the parental strain. This is supported by studies in *E. coli* in which a phenotype for Δ*yehUT* mutant was also not detected [3,5]. 

The *yehUT* operon of *S.* Typhi may be undergoing either adaptive evolution due to host or environmental stresses or possibly a form of relaxed evolution as evidenced by the accumulation of a high number of non-synonymous SNPs. Further investigation into the biological complexity of these individual SNPs within *S.* Typhi needs to be undertaken to answer these questions more fully.

## Supporting Information

Figure S1 **Resistance to killing by human serum of (**A**) *Salmonella* Typhimurium ST4/74, *S*. Typhimurium ST4/74 Δ*yehUT* and *S*. Typhimurium *∆yehUT::cat*** :***: yehUT::aph*, and (B) *S***. **Typhi BRD 948, *S*. Typhi Δ*yehUT* and *S*. Typhi *∆yehUT ::cat* pWKS30::*yehUT::aph*.**
Deletion of *yehUT* had no detectable impact on the resistance to antibody-mediated, complement dependent killing of either *S*. Typhimurium (A) or *S*. Typhi (B) (*p*>0.05 for all time points). Complementation of the *yehUT* locus for both *S*. Typhimurium and *S*. Typhi mutant derivatives also had no detectable effect on resistance to serum killing. Data for each line represent the mean of the results from 10 healthy adult donors done in triplicate. The initial concentration of bacteria in the assay is approximately 1x10^6^ cfu/ml. Negative values show a decrease in the number of viable bacteria over time. Error bars represent SEM.
(EPS)

Figure S2 **Comparison of the ability of (**A**) *S*.** **Typhi BRD 948 and *S*. Typhi Δ*yehUT*, and (B) *S*. Typhimurium ST4/74 and *S*. Typhimurium ST4/74 Δ*yehUT* to invade HEp-2 cells**.
For each isloate three experiments were conducted with bacteria grown by shaking in low salt LB for 6 hours at 37°C followed by inoculating 0.5mls of this culture into 45mls fresh low salt LB in a 50ml falcon tube. These samples were left to grow statically overnight at 37°C. Each column represents the mean and SEM of numbers of viable bacteria recovered from three separate wells. In (A) the data was normalized to *S*. Typhi BRD948 and the invasiveness of *S*. Typhi Δ*yehUT* was not obviously affected (p=0.38). In (B) the data was normalized to *S*. Typhimurium ST4/74 and the invasiveness of *S*. Typhimurium Δ*yehUT* was also not obviously affected (p=0.33).
(EPS)

Figure S3 **Vi phage assays of *S*.** **Typhi BRD948 and *S*. Typhi Δ*yehUT***.
Plaque morphology and plaque counts (pfu/ml) were assessed after overnight Vi phage types II and VII infections of both *S*. Typhi BRD948 and *S*. Typhi BRD948 *yehUT*. There was no significant difference between *S*. Typhi and *S*. Typhi *yehUT* in terms of plaque morphology or counts for either Type II (p=0.41) or Type VII (p=0.66) phage infections.
(EPS)

Figure S4 **Mixed competitive inhibition mouse experiment of *S*. Typhimurium ST4/74 and *S*. Typhimurium ∆*yehUT***.
The competitive index (CI) ratio of output (∆*yehUT* mutant/*S*. Typhimurium ST4/74) / input (∆*yehUT* / *S*. Typhimurium ST4/74) was calculated for bacterial colony-forming units (CFU) in tissue from mesenteric lymph nodes (MLN), cecum, ileum, spleen and liver of five C57BL/6 mice orally inoculated with 10^8^ CFU of *S*. Typhimurium ST4/74 and the ∆*yehUT* derivative of this parental isolate. This experiment was performed twice. Mean (central bars) and 95% confidence intervals (error bars) are shown. Results showed that there were no significant differences in bacterial counts between *S*. Typhimurium ST4/74 and *S*. Typhimurium ∆*yehUT* in the MLN (p= 0.5), cecum (p=0.91), ileum (p=0.50), spleen (p=0.04) and liver (p= 0.004).
(EPS)

Figure S5 **Cecal bacterial counts of *S*. Typhimurium ST4/74, *S*. Typhimurium ∆*yehUT* and *S*. Typhimurium ∆*yehUT::cat*** :***: yehUT::aph*, in the streptomycin-pretreated colitis mouse experiment**.
CFUs in tissue from cecum of five C57BL/6 mice pretreated with 50mg of streptomycin and then 24 hours later orally inoculated with 10^3^ CFU of either (a) *S*. Typhimurium ST4/74, (b) *S*. Typhimurium ∆*yehUT* or (c) *S*. Typhimurium ∆*yehUT::cat*:*: yehUT::aph*. This experiment was performed three times. Mean (central bars) and SEM (error bars) are shown. The results showed that there were no significant differences between *S*. Typhimurium ST4/74 and the ∆*yehUT* derivative (p=0.16) and between ∆*yehUT* and ∆*yehUT::cat*:*: yehUT::aph* (p=0.62).
(EPS)

Figure S6 **Histopathological scoring of cecal segments of *S*. Typhimurium ST4/74, *S*. Typhimurium ∆*yehUT* and *S*. Typhimurium ∆*yehUT::cat*** :***: yehUT::aph* in the streptomycin-pretreated colitis mouse experiment**.
Histopathological scoring of hematoxylin and eosin stained mouse cecal segments after five C57BL/6 mice were pretreated with 50mg of streptomycin and then 24 hours later orally inoculated with 10^3^ CFU of either *S*. Typhimurium ST4/74, *S*. Typhimurium ∆*yehUT* or *S*. Typhimurium ∆*yehUT::cat*:*: yehUT::aph*. This experiment was performed three times. Scoring of intestinal inflammation was conducted by a Consultant Histopathologist and was as follows: submucosal edema: mild-1, moderate-2, severe-3; submucosal inflammation: mild-1, moderate-2, severe-3; mucosal inflammation: mild-1, moderate-2, severe-3; crypt abscesses: absent-0, present (occasional/mild)-1, present (moderate/severe)-2; mucosal ulceration: absent-0, present (focal/mild)-1, present (moderate/severe)-2. There were no significant differences in total pathological scores between the three groups.
(EPS)

Figure S7 **qRT-PCR of inflammatory cytokines from cecal segments of *S*.Typhimurium ST4/74, *S*. Typhimurium ∆*yehUT* and *S*. Typhimurium ∆*yehUT::cat*** :***: yehUT::aph* in the streptomycin-pretreated colitis mouse experiment**.
qRT-PCR of inflammatory cytokines from mouse cecal segments after four groups of five C57BL/6 mice were pretreated with 50mg of streptomycin and then 24 hours later either not infected with bacteria (a=naive) or orally inoculated with 10^3^ CFU of (b) *S*. Typhimurium ST4/74, (c) *S*. Typhimurium ∆*yehUT* or (d) *S*. Typhimurium ∆*yehUT::cat*:*: yehUT::aph*. This experiment was performed twice. Cytokine levels of IL-1, IL-6, KC, Mip-2, TNF-α, IFN-γ, iNOS, and Lys6-G were measured using qRT-PCR and the relative gene expression determined by normalization to Gapdh. The mean (central bars) and SEM (error bars) are shown. The results showed that there were no significant differences between (b) *S*. Typhimurium ST4/74 and (c) ∆*yehUT* for IL-1 (p=0.74), IL-6 (p=0.5), KC (p=0.67), Mip-2 (p=0.35), TNF-α (p=0.58), IFN-*g* (0.58), iNOS (p=0.31) and Lys6-G (p=0.89).
(EPS)

File S1 **Supporting Information Tables: Supplementary data for Characterization of the *yehUT* two-component regulatory system of *Salmonella enterica* serovar Typhi and Typhimurium.** Table S1. Primers used to construct the *yehUT* mutants of *S*. Typhi BRD948 and *S*. Typhimurium ST4/74. Primers used in this study. The nucleotides denoted in small letters are homologous to regions adjacent to the target gene and the nucleotides in capital letters are homologous to the template plasmids, pKD3 or pKD4, carrying antibiotic resistance genes that are flanked by FRT (FLP recognition target) sites; Table S2. Primer used to construct the complement mutants of *S*. Typhi BRD948 *ΔyehUT* and *S*. Typhimurium ST4/74 *ΔyehUT*. Primers used in this study. The nucleotides denoted in small letters are homologous to regions adjacent to the target gene and the nucleotides in capital letters are homologous to the template plasmids carrying antibiotic resistance genes that are flanked by FRT (FLP recognition target) sites; Table S3. Primers used in the qRT-PCR gene expression experiment of *S*. Typhi BRD948 *ΔyehUT* mutant; Table S4. Primers used in the qRT-PCR gene expression experiment of *S*. Typhimurium *ΔyehUT* mutant; Table S5. Primers used in the mouse cecal qRT-PCR cytokine experiment; Table S6. qRT-PCR analyses of differentially expressed genes of the S. Typhi Δ*yehUT* mutant and *S*. Typhimurium Δ*yehUT* mutant; Table S7. SNPs identified by informatics analysis of the *yehUT* genes of the S. Typhimurium SL1344 genome (Accession number: FQ312003). Coordinates correspond to *S*. Typhi CT18 finished genome sequence (Accession number: AL513382).
(DOCX)
