# Supplementary material for: Characterization of the yehUT Two-Component Regulatory System of Salmonella enterica Serovar Typhi and Typhimurium
Source: PLoS One. 2013 Dec 30;8(12):e84567. doi: 10.1371/journal.pone.0084567 (PMC3875573; doi:10.1371/journal.pone.0084567)
Supplement: File S1 — Supporting Information Tables: Supplementary data for Characterization of the yehUT two-component regulatory system of Salmonella enterica serovar Typhi and Typhimurium. Table S1. Primers used to construct the yehUT mutants of S. Typhi BRD948 and S. Typhimurium ST4/74. Primers used in this study. The nucleotides denoted in small letters are homologous to regions adjacent to the target gene and the nucleotides in capital letters are homologous to the template plasmids, pKD3 or pKD4, carrying antibiotic resistance genes that are flanked by FRT (FLP recognition target) sites; Table S2. Primer used to construct the complement mutants of S. Typhi BRD948 ΔyehUT and S. Typhimurium ST4/74 ΔyehUT. Primers used in this study. The nucleotides denoted in small letters are homologous to regions adjacent to the target gene and the nucleotides in capital letters are homologous to the template plasmids carrying antibiotic resistance genes that are flanked by FRT (FLP recognition target) sites; Table S3. Primers used in the qRT-PCR gene expression experiment of S. Typhi BRD948 ΔyehUT mutant; Table S4. Primers used in the qRT-PCR gene expression experiment of S. Typhimurium ΔyehUT mutant; Table S5. Primers used in the mouse cecal qRT-PCR cytokine experiment; Table S6. qRT-PCR analyses of differentially expressed genes of the S. Typhi ΔyehUT mutant and S. Typhimurium ΔyehUT mutant; Table S7. SNPs identified by informatics analysis of the yehUT genes of the S. Typhimurium SL1344 genome (Accession number: FQ312003). Coordinates correspond to S. Typhi CT18 finished genome sequence (Accession number: AL513382). (DOCX) [file pone.0084567.s008.docx]

**Supplementary data**

**Table S1. Primers used to construct the *yehUT* mutants of *S.* Typhi BRD948 and *S.* Typhimurium ST4/74.**

| **Oligonucleotide reference** | **Sequence** | **Mutant** |
| --- | --- | --- |
| ***S.* Typhi BRD948** | | |
| RKS593-forward | atgtacgagtttaatctggtgttgctgctgcttcagcagatgtgcGTGTAGGCTGGAGCTGCTTCG | Δ*yehU* |
| RKS592-reverse | ccgcattatcttcaatatctaacatcaaatgctgcccttcgcgccgCATATGAATATCCTCCTTAG |  |
| RKS566-forward | tttacccgaattacattacgacttccactggaggaggacgcatgaGTGTAGGCTGGAGCTGCTTCG | Δ*yehT* |
| RKS594-reverse | gttcttcaaaggctttgatggcgtattcgtcaaacgcggttaaaaaCATATGAATATCCTCCTTAG |  |
| RKS593-forward | atgtacgagtttaatctggtgttgctgctgcttcagcagatgtgcGTGTAGGCTGGAGCTGCTTCG | Δ*yehUT* |
| RKS594-reverse | gttcttcaaaggctttgatggcgtattcgtcaaacgcggttaaaaaCATATGAATATCCTCCTTAG |  |
| RKS570-forward | gtaagccgtcgctatctgaaaagtttaaaagaggcgattggcctgGACTACAAAGACCATGACGG | Δ*yehT* FLAG |
| RKS569-reverse | ccttcggtcgtttctatggcaaaacgatattctaacagtcttttaATATGAATATCCTCCTTAG |  |
| ***S.* Typhimurium ST4/74** | | |
| RKS593-forward | atgtacgagtttaatctggtgttgctgctgcttcagcagatgtgcGTGTAGGCTGGAGCTGCTTCG | Δ*yehU* |
| RKS595-reverse | ccgcattatc**c**tcaatatctaacatcaaatgctgcccttcgcgccgCATATGAATATCCTCCTTAG |  |
| RKS566-forward | tttacccgaattacattacgacttccactggaggaggacgcatgaGTGTAGGCTGGAGCTGCTTCG | Δ*yehT* |
| RKS594-reverse | gttcttcaaaggctttgatggcgtattcgtcaaacgcggttaaaaaCATATGAATATCCTCCTTAG |  |
| RKS593-forward | atgtacgagtttaatctggtgttgctgctgcttcagcagatgtgcGTGTAGGCTGGAGCTGCTTCG | Δ*yehUT* |
| RKS594-reverse | gttcttcaaaggctttgatggcgtattcgtcaaacgcggttaaaaaCATATGAATATCCTCCTTAG |  |
| RKS570-forward | gtaagccgtcgctatctgaaaagtttaaaagaggcgattggcctgGACTACAAAGACCATGACGG | Δ*yehT* FLAG |
| RKS569-reverse | ccttcggtcgtttctatggcaaaacgatattctaacagtcttttaATATGAATATCCTCCTTAG |  |
| **Check primers for *S.* Typhi BRD948 and *S.* Typhimurium ST4/74** | | |
| RKS573-forward | aagtgatcgtattcacgaattc | Δ*yehU* check |
| RKS567-reverse | tgtagcgcacgctacgcag |  |
| RKS568-forward | tggcgatgattatggtattagc | Δ*yehT* check |
| RKS567-reverse | tgtagcgcacgctacgcag |  |
| RKS573-forward | aagtgatcgtattcacgaattc | Δ*yehUT* check |
| RKS567-reverse | tgtagcgcacgctacgcag |  |
| RKS568-forward | tggcgatgattatggtattagc | Δ*yehT* FLAG check |
| RKS567-reverse | tgtagcgcacgctacgcag |  |

**Table S2. Primer used to construct the complement mutants of S. Typhi BRD948 *ΔyehUT* and *S.* Typhimurium ST4/74 *ΔyehUT.***

| **Oligonucleotide reference** | **Sequence** | **Mutant** |
| --- | --- | --- |
| ***S.* Typhi BRD948** | | |
| RKS662-forward | cgctGAGCTCcgcagaatatcgttactcagc | *S.* Typhi *∆yehUT* ::*cat* pWKS30::*yehUT::aph* |
| RKS663-reverse | cgcGGATCCcatcgtttcacccttgctcg |  |
| ***S.* Typhimurium ST4/74** | | |
| vkw3-forward | gaacacgcggcgaagtaattctgcggtattgttcccggcagcgtcGTGTAGGCTGGAGCTGCTTCG | *S.* Typhimurium *∆yehUT::cat:: yehUT::aph* |
| vkw4-reverse | atcatagaatcaattagtataataatgcgttttcccggtcagacagCATATGAATATCCTCCTTAG |  |
| **Check primers for *S.* Typhimurium ST4/74** | | |
| Recon1for-forward | ctgccgcacaagcttctgtgttac | *S.* Typhimurium *∆yehUT::cat:: yehUT::aph* |
| Recon2Rev-reverse | ttcctaccatctccagtccactga |  |

**Table S3. Primers used in the qRT-PCR gene expression experiment of *S.* Typhi BRD948 *ΔyehUT* mutant**

| **Oligonucleotide reference** | **Sequence** | **Gene name** |
| --- | --- | --- |
| t4582-forward | aatcatcggtgctttctgcctctc | *cstA1* |
| t4582-reverse | cgcgtcgggtcgagtttcat |  |
| t4581-forward | aggcggcaaaaatgttgattgg | *t4581* |
| t4581-reverse | aacagcgcataccgcctttg |  |
| t4580-forward | accccgattgcagttaccctactt | *t4580* |
| t4580-reverse | cgcgatcgccaatcagttga |  |
| t4221-forward | acctgtccaggtggggagcat | *t4221* |
| t4221-reverse | ctccccggacgatgtcgtaa |  |
| t4220-forward | ttgagatatctgacaatgca | *t4220* |
| t4220-reverse | gcaacttttaacagataatc |  |
| t2795-forward | attcaggaaaaacggagcgagc | *spaM* |
| t2795-reverse | tgactcggcctcttcctgctgta |  |
| t2794-forward | gcaggatgaggttggcggttt | *spaN* |
| t2794-reverse | gtcgccatgccgccagtaat |  |
| t2796-forward | cccacaaaaaataaccggccc | *spaI* |
| t2796-reverse | taaggctcagcacggtgcgt |  |
| t1830-forward | tgcttttgggaggtaattggaaaac | *pipB* |
| t1830-reverse | ttttcggatgctgctccaca |  |
| t1038-forward | aaaatttcgatgggtcgttctcg | *t1038* |
| t1038-reverse | tggcgcaaataccgctgaaa |  |
| t3592-forward | gtggccgcggataacaagaga | *yiiD* |
| t3592-reverse | ccatcccgttccgacccttt |  |
| t3505-forward | ctggcctgctgacgcaaaac | *ppc* |
| t3505-reverse | caccagcggtacgccgaaac |  |
| t1321-forward | gcaaaaaggctggaaattctgacg | *nth* |
| t1321-reverse | agtttggcggtcgccttgtt |  |
| t3408-forward | gtacagcagcgcgttgaagaaga | *t3408* |
| t3408-reverse | aagctgcacacccggaatgg |  |
| t4303-forward | ttcaccgaggaagcgcatctg | *sopE* |
| t4303-reverse | agcttcacgggtctggctgg |  |
| recA-forward | gaacacgcgcttgaccctgtt | *recA* |
| recA-reverse | ccgctacggagtcgaccaca |  |

**Table S4. Primers used in the qRT-PCR gene expression experiment of *S.* Typhimurium *ΔyehUT* mutant**

| **Oligonucleotide reference** | **Sequence** | **Gene name** |
| --- | --- | --- |
| SL4463-forward | ctcgcggtgctggcattaatc | *cstA1* |
| SL4463-reverse | caatcacgccgccgaagtaa |  |
| 05703-forward | cacccagggtatgtgcttgaat | *SL1344_SPAB_05703* |
| 05703-reverse | acccagcttcggctgataattaa |  |
| SL0588-forward | cgctggtctccgcctgtattatc | *SL1344_0588 (cstA2)* |
| SL0588-reverse | ccgcctgcgcgggagataat |  |
| SL4462 -forward | aatatctcggccaggcggcaaaaatgtt | *SL1344_4462* |
| SL4462-reverse | ttaacaacagcgcataccgcctttgc |  |
| recA-forward | ggctatcgacgaaaacaaacagaaag | *recA* |
| recA-reverse | ttccggcccgtaaatttcga |  |

**Table S5. Primers used in the mouse cecal qRT-PCR cytokine experiment**

| **Oligonucleotide reference** | **Sequence** | **Gene name** |
| --- | --- | --- |
| Gapdh-forward | tgtgtccgtcgtggatctga | *Gapdh* |
| Gapdh-reverse | caccaccttcttgatgtcatcatac |  |
| Gapdh-probe | tgccgcctggagaaacctgcc |  |
| IFNg-forward | cagcaacagcaaggcgaaa | *IFNg* |
| IFNg-reverse | ctggacctgtgggttgttgac |  |
| IFBg-probe | aggatgcattcatgagtattgccaagtttga |  |
| TNFa-forward | catcttctcaaaattcgagtgacaa | *TNFa* |
| TNFa-reverse | ccagctgctcctccacttg |  |
| TNFa-probe | cctgtagcccacgtcgtagcaaacca |  |
| IL-6-forward | acaagtcggaggcttaattacacat | *IL-6* |
| IL-6-reverse | ttgccattgcacaactcttttc |  |
| IL-6-probe | ttctctgggaaatcgtggaaatg |  |
| Ly6g-forward | tgccccttctctgatggatt | *Ly6g* |
| Ly6g-reverse | tgctcttgactttgcttctgtga |  |
| Ly6g-probe | tgcgttgctctggagatagaagttattgtggact |  |
| iNOS-forward | tgcatcggcaggatcca | *iNOS* |
| iNOS-reverse | aacatttcctgtgctgtgctaca |  |
| iNOS-probe | cctgcaggtctttgacgctcggaa |  |
| KC/CXCL1-forward | gctcccttggttcagaaaattg | *KC/CXCL1* |
| KC/CXCL1-reverse | catcagagcagtctgtcttctttctc |  |
| KC/CXCL1-probe | caaaagatgctaaaaggtgtc |  |
| MIP-2/CXCL2-forward | ccctggttcagaaaatcatcca | *MIP-2/CXCL2* |
| MIP-2/CXCL2-reverse | gctcctcctttccaggtcagt |  |
| MIP-2/CXCL2-probe | aagatactgaacaaaggcaa |  |
| IL1b-forward | ccaaaagatgaagggctgctt | *IL-1b* |
| IL1b-reverse | tgctgctgcgagatttgaag |  |
| IL1b-probe | caaacctttgacctgggctgtcctga |  |

**Table S6. qRT-PCR analyses of differentially expressed genes of the *S.* Typhi Δ*yehUT* mutant and  *S.* Typhimurium Δ*yehUT* mutant**

| **Genes differentially regulated on DNA microarray analysis**  **(p< 0.05)** | **Fold change relative to wild type strain *S.* Typhi BRD948** | |
| --- | --- | --- |
|  | ***S.* Typhi Δ*yehUT*** | ***S.* Typhi *∆yehUT* ::*cat* pWKS30::*yehUT::aph*** |
| *pipB* | 0.286 | 1.880 |
| *spaM* | 0.312 | 1.110 |
| *t4580* | 0.117 | 1.589 |
| *t4220* | 0.373 | 1.360 |
| *yiiD* | 0.532 | 0.928 |
| *spaI* | 0.234 | 2.746 |
| *spaN* | 0.315 | 1.391 |
| *Ppc* | 0.289 | 1.454 |
| *t4221* | 0.304 | 2.118 |
| *cstA1* | 0.005 | 1.690 |
| *Nth* | 2.576 | 1.030 |
| *t1038* | 2.042 | 1.253 |
| *t4581* | 0.123 | 1.170 |
| *sopE* | 0.333 | 0.977 |
| *t3408* | 0.462 | 1.608 |
| **Genes differentially regulated on DNA microarray analysis**  **(p< 0.05)** | **Fold change relative to wild type strain *S.* Typhimurium ST4/74** | |
|  | ***S.* Typhimurium Δ*yehUT*** | ***S.* Typhimurium *∆yehUT::cat:: yehUT::aph*** |
| *cstA1* | 0.024 | 0.753 |
| *cstA2* | 1.509 | 1.395 |
| *SL1344_05703* | 0.049 | 0.834 |
| *SL1344_4462* | 0.235 | 0.745 |

**Table S7. SNPs identified by informatics analysis of the *yehUT* genes of the *S.* Typhimurium SL1344 genome (Accession number: FQ312003). Coordinates correspond to *S.* Typhi CT18 finished genome sequence (Accession number: AL513382).**

| **SNP position** | **Coding region** | **Gene start** | **Gene stop** | **Gene strand** | **Gene ID** | **Gene name** | **SNP class** | **Ancestral allele** | **Derived allele** |
| --- | --- | --- | --- | --- | --- | --- | --- | --- | --- |
| 2219521 | protein coding | 2219326 | 2220045 | - | STY2388 | *yehT* | Non-synonymous | A | G |
| 2220188 | protein coding | 2220042 | 2221727 | - | STY2389 | *yehU* | Non-synonymous | T | C |
| 2220487 | protein coding | 2220042 | 2221727 | - | STY2389 | *yehU* | Non-synonymous | G | A |
| 2220583 | protein coding | 2220042 | 2221727 | - | STY2389 | *yehU* | Non-synonymous | A | G |
| 2220671 | protein coding | 2220042 | 2221727 | - | STY2389 | *yehU* | Non-synonymous | T | C |
